# International survey on diagnosis and management of hypotension in extremely preterm babies

**DOI:** 10.1007/s00431-013-2251-9

**Published:** 2014-01-04

**Authors:** Zbynek Stranak, Jana Semberova, Keith Barrington, Colm O’Donnell, Neil Marlow, Gunnar Naulaers, Eugene Dempsey, On behalf of the HIP consortium

**Affiliations:** 1 Institute for the Care of Mother and Child, Third Faculty of Medicine, Charles University, Podolske Nabrezi 157, 147 00 Prague 4, Czech Republic; 2 Centre Hospitalier Universitaire Sainte-Justine, 5200, rue Bélanger East, Montreal, H1T 1C9 Quebec Canada; 3 National Maternity Hospital, Holles Street, Dublin 2, Ireland; 4 University College London, 6-96 Chenies Mews, WC1E 6HX London, UK; 5 Katholieke Universiteit Leuven, Oude Markt 13, 3000 Leuven, Belgium; 6 Department of Paediatrics and Child Health, Neonatal Intensive Care Unit, Wilton Cork, Ireland

**Keywords:** Diagnosis, Extremely low gestational age, Hypotension, Survey, Treatment

## Abstract

Hypotension is a commonly diagnosed and treated complication of extremely low gestational age newborns (ELGAN), but enormous variation in diagnosis, management and clinical practice has been documented. We sought to evaluate practice regarding the management of hypotension in ELGANs and developed a web-based questionnaire addressing diagnosis, intervention thresholds and modes of treatment of hypotension in ELGANs. We received 216 completed questionnaires from respondents in 38 countries. Most responses (83 %) were from specialist units where, together, over 26,000 very low birth weight (VLBW) infants are cared for annually. The majority (73 %) defined hypotension as a mean blood pressure (BP) in mmHg less than the gestational age in weeks. Sixty percent assessed the circulation with additional methods; echocardiography was the most commonly used (74 %), with left ventricular output and fractional shortening the two most common measurements made. The majority (85 %) used volume administration as the initial intervention. Dopamine was the inotrope most commonly used initially (80 %). If the initial inotrope therapy failed, dobutamine was the most popular second-line treatment (28 %). Delayed cord clamping was used at 51 % of the centres. *Conclusion*: The definition of hypotension in ELGANs continues to follow traditional standards. Functional echocardiography is now used to assess the circulation at many centres. Volume expansion and dopamine remain the most frequently used therapies.

**Electronic supplementary material:**

The online version of this article (doi:10.1007/s00431-013-2251-9) contains supplementary material, which is available to authorized users.

## Introduction

Hypotension is commonly diagnosed and treated in extremely low gestational age newborns (ELGANs, less than 28 weeks of gestation), but large variations in diagnosis, management and clinical practice have been previously documented [3, 9]. However, many ELGANs with low numerical blood pressure (BP) values may have adequate systemic perfusion [17].

Previously, many clinicians relied solely on absolute BP values to guide intervention, and, in some instances, single absolute mean BP values were chosen over a wide range of gestational ages [11]. In accordance with a recommendation of the Joint Working Group of the British Association of Perinatal Medicine [5], a mean arterial BP less than the infants GA has been widely used to diagnose hypotension [3]. This method of diagnosis has no empirical evidence to support it but appears to be the historical standard largely by virtue of its simplicity and clinical availability [5]. There is no validated clinical scoring system available to assess poor perfusion associated with apparently low blood pressure values in the preterm infant. A number of indirect measures applicable at the bedside include physical and biochemical findings [1]. There has been increased use of ancillary methods to assess perfusion in the setting of newborn hypotension, including bedside echocardiography [8, 16]. Enormous variations in the treatment of hypotension have been documented, which have not been shown to affect long-term outcomes [17]. The aim of this survey was to determine current strategies for diagnosing and treating hypotension in the first 72 h in extremely preterm infants internationally.

## Methods

A web-based questionnaire addressing diagnostic and management criteria for hypotension in ELGANs was developed by the Hypotension in the Preterm (HIP) consortium (http://www.hip-trial.com/hip-home/). This consortium comprises neonatologists, pharmacologists and neurophysiologists, and is funded by the European Union through the Seventh Framework Programme (FP7). The goal of the consortium is to determine the short- and long-term outcome of extremely low gestational age newborns treated with current practices for hypotension during the transitional period after birth.

We developed a 25-item questionnaire introduced by a specific scenario, namely, a baby born at 25 weeks of gestation with a mean blood pressure of 23 mmHg 6 h after birth (see Appendix). The questions posed related to diagnosis, methods of assessment and treatment. We collected demographic and population data, as well as institutional recommendations regarding diagnostic and therapeutic approach to hypotension. Questions evaluated the current strategies for diagnosis of hypotension including clinical signs, biochemical parameters, use of functional echocardiography, near infrared spectroscopy (NIRS), electroencephalography (EEG) and additional methods for circulatory assessment (e.g. central venous pressure, mixed venous saturation and pulsatility index). Specific management-related questions addressed which agent was used first after hypotension has been diagnosed (volume and inotrope or steroids). If volume therapy was used, we asked respondents to specify the amount and type of fluid. Clinicians’ preferences for various inotropes were investigated. Particular agents and their dosing regimens were addressed. Participants were asked whether they changed their approach to hypotension management in the presence of a patent ductus arteriosus that they considered haemodynamically significant. Other questions related to whether delayed cord clamping was applied routinely and whether strategies, including ‘permissive hypotension’ [2]—i.e. ‘watchful waiting’ in preference to immediate intervention for infants with numerically low BP values in the absence of other signs of poor perfusion—were used.

The survey was distributed in cooperation with the neonatologists involved in the HIP consortium, who coordinated distribution at national level within their own countries to all Level II and III units. It was also distributed at the European Society for Paediatric Research meeting in Istanbul, Turkey, October 2012. The questionnaire was anonymous, and participation was voluntary. Returned completed questionnaires from each physician were taken as consent to participate in the study.

Data were analysed with SPSS statistical software version 19 (IBM, Armonk NY, USA). Following descriptive analysis, confidence intervals of proportions were calculated for dichotomous variables. Analysis of the most common hypotension treatment method was performed by using contingency tables using a sign scheme displayed for adjusted standardised residuals. The relationship of selected answers was compared among various responder groups by using three-dimensional contingency tables. Analysis of the most common treatment method was strengthened by the use of cluster analysis based on treatment method information, cross-referenced to responder group representation.

## Results

We received 221 responses and excluded 5, as data were incomplete, leaving 216 questionnaires available for analysis. Most survey respondents (83 %) were from specialist or Level III centres, and 41 % were affiliated to University hospitals caring for a combined figure of over 26,000 very low birth weight (VLBW, <1500 g) infants annually. Characteristics of the institutions from which participants responded are presented in Table 1. The response rate was 100 % for Irish, Czech and Belgian centres.

**Table 1:** Institution demographics of survey participants

Characteristic (no. of responders)		Number (%)
Level of care provided (*n* = 215)	Level I	6 (3)
	Level II	31 (14)
	Level III	178 (83)
Number of beds in NICU (*n* = 213)	<10	61 (29)
	10–29	123 (58)
	30–49	20 (9)
	>50	9 (4)
Number of VLBW infants per year (*n* = 195)	≤10	18 (9)
	11–50	42 (22)
	51–100	54 (28)
	>101	81 (42)
Type of hospital (*n* = 215)	University hospital	87 (41)
	Paediatric hospital	29 (14)
	General hospital	85 (40)
Institutional diagnostic recommendation (*n* = 205)	Yes	125 (61)
Institutional treatment recommendation (*n* = 203)	Yes	132 (65)

Recommendations for diagnosis and treatment of hypotension were established in 61 and 65 % of all centres, respectively. Hypotension was defined as a mean BP in mmHg less than the GA in weeks by 73 % of respondents. Other criteria including predefined percentile or specific limits believed to be associated with poor outcome were used significantly less often (10 and 4 %, respectively); 12 % reported using a combination of these criteria.

### Diagnostic methods of poor perfusion

**Fig. 1:**
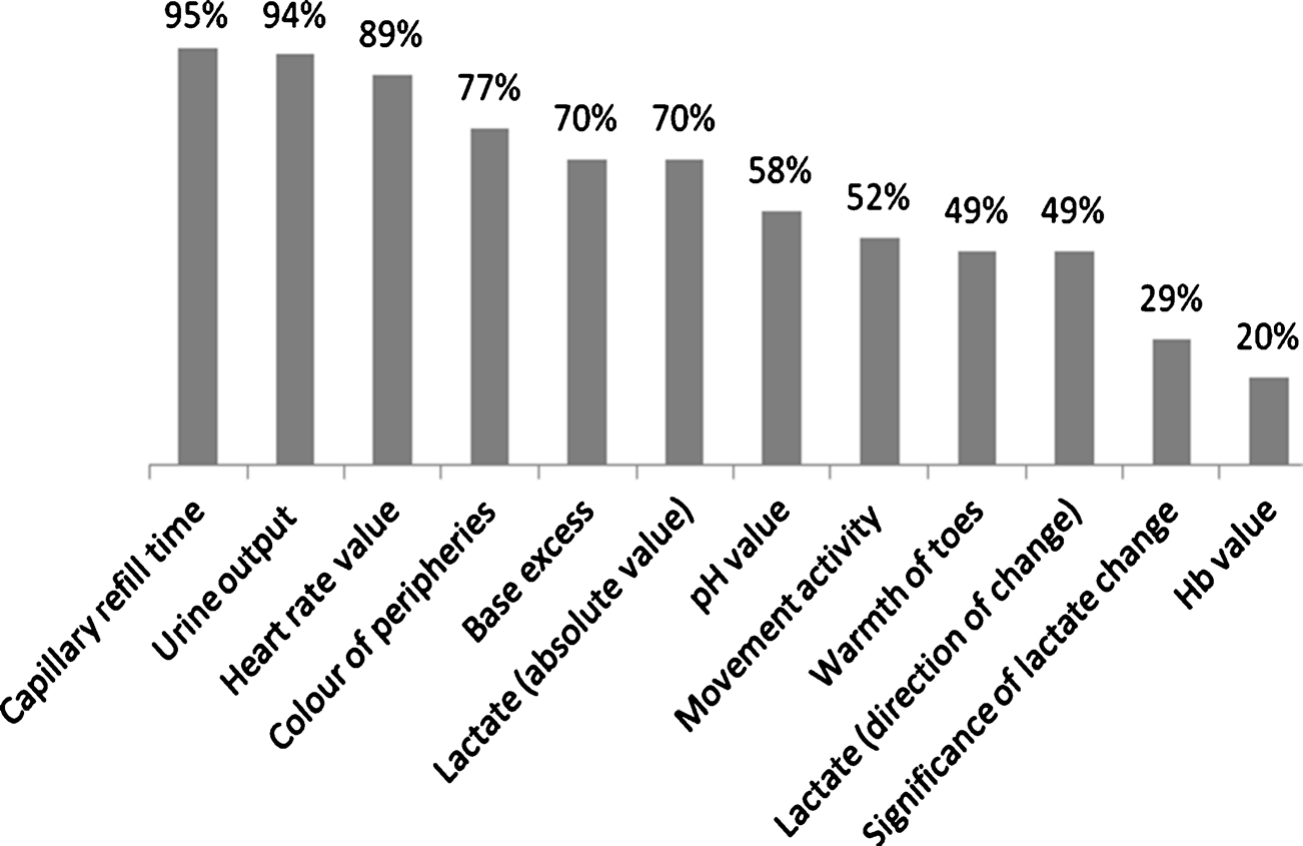
Proportion of clinical and laboratory methods used to evaluate perfusion

All respondents assessed perfusion clinically (Fig. 1), with measurement of capillary refill time (CRT) on the chest the preferred method (76 %). This was considered pathological if it is more than 3 s by 59 % of respondents. Laboratory methods were used by 75 %, the commonest being both acid base measurement (70 %) and lactate analysis (70 %). Serum biomarkers of myocardial dysfunction—including pro-Brain Natriuretic Peptide, Troponin T and Troponin I—were documented by 28 % of respondents. Ancillary methods of assessment were used by 60 % of participants (Table 2). Of these, the most frequently used was echocardiography (74 %). Table 2 highlights the results of the most commonly reported echo measurements. The predominant measurement performed was left ventricular output (LVO) followed by fractional shortening of left ventricle. Right ventricular output (RVO) or superior vena cava (SVC) flow was used less frequently (34 and 38 %, respectively).

**Table 2:** Ancillary investigations used to evaluate poor perfusion (180 respondents)

Measurement	Number (%)	95 % confidence interval	
		Lower bound	Upper bound
ECHO use	134 (75)	68	81
Left cardiac output	87 (65)	57	73
Right cardiac output	45 (34)	26	42
Fractional shortening of left ventricle	67 (50)	41	59
SVC flow	51 (38)	30	46
Perfusion index	31 (17)	12	23
Temperature measure	85 (47)	40	55
CVP measure	22 (12)	7	17
Mixed venous O2	18 (10)	6	14
EEG/aEEG	6 (3)	1	6
NIRS measurement	27 (15)	10	20

*ECHO* echocardiography, *SVC* superior vena cava, *CVP* central venous pressure, *EEG* Electroencephalography, *NIRS* near infrared spectroscopy

### Therapeutic approaches to hypotension

Overall 85 % reported giving a fluid bolus as their first treatment, with the majority (93 %) administering crystalloid. The initial amount administered was 10 ml/kg by 82 % of respondents. The total volume given before using another agent was 20 ml/kg by 59 %, 30 ml/kg by 14 % and >30 ml/kg in 13 % of centres. Dopamine was the most commonly used first-line inotrope (80 %), used alone (62 %) or in combination with dobutamine (18 %). The median starting dose of dopamine was 5 mcg/kg/min, and median maximum dose was 20 mcg/kg/min. If the BP did not increase with the initial inotrope infusion, dobutamine with dopamine was the most popular second-line treatment (28 %). However, there was great variation in the choice of the second agent used (Table 3). Seventy-five percent of respondents altered the therapeutic regime when managing low BP in the presence of a patent ductus arteriosus (PDA) considered to be haemodynamically significant. In this situation, indomethacin or ibuprofen was given by 77 %, with fluid restriction or avoidance of volume administration less often used (46 and 29 %, respectively).

**Table 3:** Choice of inotrope intervention for hypotension (188 respondents)

	Number (%)	95 % confidence interval	
		Lower bound	Upper bound
First-line treatment			
Dopamine	116 (62)	55	69
Dobutamine	34 (18)	13	24
Dopamine and dobutamine	33 (18)	12	23
Epinephrine	4 (2)	0	4
Norepinephrine	1 (1)	0	2
Second-line treatment			
Dopamine	14 (7)	4	11
Dobutamine	42 (22)	16	28
Dopamine and dobutamine	52 (28)	21	34
Epinephrine	32 (17)	12	22
Norepinephrine	18 (10)	5	14
Steroids	18 (10)	5	14
Milrinone	2 (1)	0	3
Notropes and steroids	11 (6)	3	9

### Therapeutic regimens

Variables were subjected to the Twostep cluster analysis to identify respondent groups who follow a similar course of treatment. The algorithm identified four different clusters, and the results show that the most prevalent approach to the hypotension therapy in ELGANs was primary volume administration followed by inotrope treatment with dopamine. Responder distribution in the particular clusters was compared with the number of intensive care beds, number of VLBW patients, type of institution, existence of institutional guidelines and the region. None of the above mentioned variables, except the region, were found to have similar cluster representation. Cluster variation in different region is shown in Table 4.

**Table 4:**
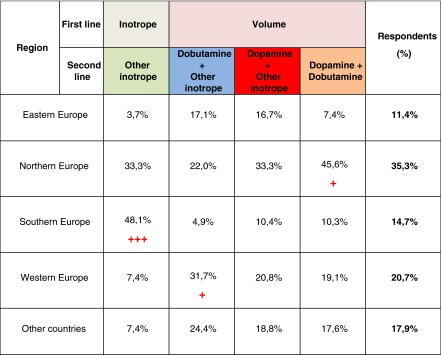
Practice variation in different region

TwoStep Cluster Analysis and Crosstabulation According Region. Variables from the survey questions addressing treatment regiment were subjected to the TwoStep Cluster analysis to identify responder groups who follow similar course of treatment. The algorithm identified four different clusters as demonstrated by coloured columns. Cluster distribution in different regions was compared using *χ* ^2^-test (asymptotic, exact and Monte Carlo, respectively). Significant differences among clusters are expressed as sign scheme. Level of significance 95 %, respectively 99,9 % is expressed as +(*p* < 0,05) and +++(*p* < 0,001), respectively

Eastern Europe: Czech Republic, Slovakia, Poland, Ukraine, Russia Northern Europe: United Kingdom, Ireland, Denmark, Norway, Sweden, Iceland, Latvia, Finland Southern Europe: Spain, Greece, Portugal, Italy, Turkey, Croatia, Slovenia Western Europe: Belgium, Germany, Netherlands, France, Switzerland and Others countries: United States, Saudi Arabia, Australia, New Zealand, South Africa, Brazil, Egypt, Israel, Japan, Canada, Lebanon, Kuwait, United Arab Emirates

Finally, the concept of ‘permissive hypotension’—not intervening when the BP is lower than previously accepted normative values in the absence of any signs of poor perfusion—appears to be an approach that many (80 %) of the respondents stated that they would consider using.

## Discussion

This survey, conducted primarily in Europe, highlights a number of diagnostic and therapeutic issues. These results do need to be interpreted cautiously as they may be biassed and not truly representative of current practice. We are aware of the limitations of this type of study (the lack of information on hypotension duration, the lack of clear definition of permissive hypotension and good perfusion and the responses may reflect personal preference rather than unit practice). Whilst we acknowledge that a survey may not translate into what one does at the bedside and notwithstanding the above limitations, taking into account the number of responses obtained, we believe these results are representative of current management practices.

At most centres, hypotension is defined as mean BP less than GA [3]. The most common diagnostic method of assessing perfusion remains clinical evaluation, but there is increasing use of echocardiography. Though there is little published evidence of clinical benefit to date, it appears that the use of functional echocardiography is increasing. However, we were surprised that LVO rather than RVO was more commonly evaluated, given the near-universal ductal patency during this time period [16].

Lactate and base excess are frequently used in the diagnosis of poor tissue perfusion. A previous study demonstrated that there was a correlation between raised lactate and poor outcome and that the prediction of poor outcome is improved if serial values are considered. Only one study appears to have correlated lactate values with directly measured systemic blood flow, and this study noted that there was wide scatter in the data, which was improved by combining capillary filling time with the serum lactate values. Serial lactate measurement was used by one half of the respondents in our survey. The use of other specific markers of myocardial dysfunction (pro BNP, Troponin T and Troponin I) is low [6, 12].

Delayed cord clamping increases circulating blood volume and appears to lead to improved haemodynamic stability [15]. It is practised by only half of the centres surveyed. The primary approach to intervention remains administration of crystalloid in an initial dose of 10 ml/kg. There is a lack of evidence to support this widespread practice [4]. In addition, fluid bolus appears to increase mortality in older children with clinically assessed impaired perfusion [10]. Our survey identified that 15 % of respondents do not use volume administration as a primary intervention. The first-line inotropic agent in this group is usually dopamine, and this approach was more prevalent in Southern European respondents.

Dopamine remains the most commonly used inotrope therapy overall. The main reason for using dopamine is the ability to increase BP, but in preterm infants with low systemic blood flow (SBF), there is no evidence that dopamine increases systemic flow. There is some suggestion that dobutamine may be a better option in increasing and maintaining SBF [14]. Dobutamine was more often used in Western Europe. Northern European respondents use volume administration (alone or combined with inotropes) as their first-line treatment and tend to choose dopamine as the inotrope of first choice. Approximately 40 % of babies with low SBF fail to respond to these inotropes [13], and increasingly, respondents are using other agents such as epinephrine or norepinephrine. Steroids (usually hydrocortisone) were used only in 10 % of centres as the second choice of circulatory support. Use of steroids for refractory hypotension has been studied in a small number of small studies and seems to reduce the duration of other inotrope use. However, long-term safety or efficacy data are lacking [7].

Infants hypotensive according to the GA criteria but with clinical evidence of good perfusion have been shown to have outcome as good as normotensive patients, and treated low BP was associated with adverse outcome [2]. The concept of ‘permissive hypotension’—not intervening for a specific BP value (typically one less than the GA) in the absence of other signs of poor perfusion—seems to be gaining popularity as the majority of respondents (80 %) stated that they would consider using this approach.

## Conclusion

The main criteria used to commence treatment for hypotension in ELGANs remains a mean BP in mmHg less than the GA, and treatment predominantly comprises volume followed by dopamine. There appears to be increasing use of point of care echocardiography in the management of these patients, though the benefit of this or other methods of assessment have not been demonstrated. The HIP trial (http://www.hip-trial.com/)—an international, multicentre double-blind, randomised, placebo controlled trial of dopamine for treatment of mean BP less than GA in a planned sample of 830 extremely preterm infants—will provide new information in this area. The role of ancillary methods of assessment including echocardiography, near infrared spectroscopy and electroencephalograhy will be assessed in a planned subgroup of infants.

## Electronic supplementary material

Below is the link to the electronic supplementary material.

ESM 1 (PDF 230 kb)
