# Supplementary material for: International survey on diagnosis and management of hypotension in extremely preterm babies
Source: Eur J Pediatr. 2014 Jan 4;173(6):793–8. doi: 10.1007/s00431-013-2251-9 (PMC4032643; doi:10.1007/s00431-013-2251-9)
Supplement: Supplementary file 1 — (PDF 230 kb) [file 431_2013_2251_MOESM1_ESM.pdf]

1. Characteristics of your NICU (Please note that Level III relates tertiary level care)

- [ ] Level I
- [ ] Level II
- [ ] Level III

Other (please specify)

2. Characteristics of your NICU:

Total number of intensive care beds

Number of very low birth weight infants annually

3. Is your NICU part of a paediatric hospital or a general hospital? (The option will be to tick as many as suitable)

- ( ) Paediatric hospital
- ( ) General hospital
- ( ) Free standing maternity
- ( ) University hospital

4. Do you have any institutional recommendations regarding the diagnosis and treatment of hypotension in first 72 hours after delivery in the ELGAN?

	Diagnosis	Treatment
Yes	☐	☐
No	☐	☐

5. How do you evaluate poor perfusion in ELGA newborn?

- [ ] Clinical examination
- [ ] Laboratory testing
- [ ] Ancillary methods (ECHO etc.)
- [ ] All of the above

6. If you use clinical examination what parameters do you measure?

	Yes	No
Capillary refill time	☐	☐
Urine output	☐	☐
Colour of peripheries	☐	☐
Heart rate	☐	☐
Warmth of toes	☐	☐
Activity level	☐	☐

Other (please specify)

7. What capillary refill time do you consider abnormal and where do you measure it?

	Abnormal time	Place of measurement
Capillary refill time (CRT)		

8. What laboratory tests do you use to evaluate poor perfusion in ELGA newborn?

- [ ] Base excess
- [ ] Haemoglobin
- [ ] Lactate (absolute value)
- [ ] pH
- [ ] Lactate (direction of change)
- [ ] Significance of lactate change
- [ ] Other (please specify)

9. What ancillary methods do you use to evaluate poor perfusion in ELGA newborn?

- [ ] ECHO
- [ ] Mixed venous O2
- [ ] Perfusion index
- [ ] EEG/aEEG
- [ ] Temperature
- [ ] NIRS
- [ ] CVP (Central Venous Pressure)
- [ ] Other (please specify)

10. Do you measure blood markers of myocardial dysfunction?

	Answer	If "YES"
Blood markers measurement		

11. Under which circumstances do you consider ELGA newborn to have low blood pressure?

- [ ] According to a predefined percentile
- [ ] According to a mean blood pressure is less than gestational age
- [ ] According to a limit you believe to be associated with poorer outcomes
- [ ] Other (please specify)

12. If functional ECHO is performed, what parameters of low perfusion do you measure?

- [ ] Left cardiac output
- [ ] Fractional shortening of left ventricule
- [ ] Right cardiac output
- [ ] SVC flow
- [ ] Other (please specify)

13. If hypotension is determined, what initial treatment you choose?

- [ ] Volume administration
- [ ] Inotropes
- [ ] Steroids

14. If volume, what agent and amount do you use initially?

	Agent	Amount
Volumotherapy		

15. If volume, what maximum cumulative amount of volume do you use?

- [ ] 20 ml/kg
- [ ] 30 ml/kg
- [ ] 40 ml/kg
- [ ] More
- [ ] No cumulative volume

16. What inotropes do you use initially?

- [ ] Dopamine
- [ ] Dopamine and dobutamine
- [ ] Dobutamine
- [ ] Norepinephrine
- [ ] Vasopressin
- [ ] Milrinone
- [ ] Other (please specify)

17. Please specify the starting dose and maximum dose from your chosen agent.

Starting dose

Maximum dose

18. If initially inotrope therapy failed what is your second choice?

- [ ] Dopamine
- [ ] Norepinephrine
- [ ] Vasopressin
- [ ] Dopamine and dobutamine
- [ ] Dobutamine
- [ ] Epinephrine
- [ ] Milrinone
- [ ] Steroids
- [ ] Levosimendane
- [ ] Other (please specify)

19. What preparation of steroid do you administer for hypotension?

- [ ] Hydrocortisone
- [ ] Methylprednisolone
- [ ] Dexamethasone
- [ ] None

Other (please specify)

20. If you administer steroids for hypotension, please specify dose in mg/kg.

Hydrocortisone

Methylprednisolone

Dexamethasone

Other

21. Do you change your approach when you consider hemodynamically significant ductus arteriosus is present?

- ( ) Yes
- ( ) No

22. If your answer of previous question (21) is Yes, please specify.

- [ ] No volumotherapy
- [ ] Increase hematocrit
- [ ] Indomethacin/ibuprofen
- [ ] Change of ventilatory setting
- [ ] Fluid restriction

23. Do you practice delayed cord clamping?

- [ ] Yes
- [ ] No

24. Do you believe that permissive hypotension is an acceptable strategy for treating ELGA newborn infants during the first few days of life?

- ○ Yes
- ○ No

25. Please fill the country of your hospital.

[ ]
